# Feasibility of a Dynamic Web Guidance Approach for Personalized Physical Activity Prescription Based on Daily Information From Wearable Technology

**DOI:** 10.2196/resprot.3966

**Published:** 2015-06-04

**Authors:** Crystal L Coolbaugh, Stephen C Raymond Jr, David A Hawkins

**Affiliations:** ^1^ University of California - Davis Biomedical Engineering Graduate Group One Shields Avenue Davis, CA United States; ^2^ University of California - Davis Department of Computer Science One Shields Avenue Davis, CA United States; ^3^ University of California - Davis Department of Neurobiology, Physiology and Behavior Room 196 Briggs Hall Davis, CA United States

**Keywords:** exercise, Web-based interventions, activity monitoring, physical fitness, algorithms

## Abstract

**Background:**

Computer tailored, Web-based interventions have emerged as an effective approach to promote physical activity. Existing programs, however, do not adjust activities according to the participant’s compliance or physiologic adaptations, which may increase risk of injury and program attrition in sedentary adults. To address this limitation, objective activity monitor (AM) and heart rate data could be used to guide personalization of physical activity, but improved Web-based frameworks are needed to test such interventions.

**Objective:**

The objective of this study is to (1) develop a personalized physical activity prescription (PPAP) app that combines dynamic Web-based guidance with multi-sensor AM data to promote physical activity and (2) to assess the feasibility of using this system in the field.

**Methods:**

The PPAP app was constructed using an open-source software platform and a custom, multi-sensor AM capable of accurately measuring heart rate and physical activity. A novel algorithm was written to use a participant’s compliance and physiologic response to aerobic training (ie, changes in daily resting heart rate) recorded by the AM to create daily, personalized physical activity prescriptions. In addition, the PPAP app was designed to (1) manage the transfer of files from the AM to data processing software and a relational database, (2) provide interactive visualization features such as calendars and training tables to encourage physical activity, and (3) enable remote administrative monitoring of data quality and participant compliance. A 12-week feasibility study was performed to assess the utility and limitations of the PPAP app used by sedentary adults in the field. Changes in physical activity level and resting heart rate were monitored throughout the intervention.

**Results:**

The PPAP app successfully created daily, personalized physical activity prescriptions and an interactive Web environment to guide and promote physical activity by the participants. The varied compliance of the participants enabled evaluation of administrative features of the app including the generation of automated email reminders, participation surveys, and daily AM file upload logs.

**Conclusions:**

This study describes the development of the PPAP app, a closed-loop technology framework that enables personalized physical activity prescription and remote monitoring of an individual’s compliance and health response to the intervention. Data obtained during a 12-week feasibility study demonstrated the ability of the PPAP app to use objective AM data to create daily, personalized physical activity guidance, provide interactive feedback to users, and enable remote administrative monitoring of data quality and subject compliance. Using this approach, public health professionals, clinicians, and researchers can adapt the PPAP app to facilitate a range of personalized physical activity interventions to improve health outcomes, assess injury risk, and achieve fitness performance goals in diverse populations.

## Introduction

Despite an extensive history of evidence-based recommendations about the amount of physical activity needed to improve or maintain health [1-7], physical inactivity has emerged as a global health concern [8] and identification of an ideal, personalized physical activity dose remains elusive. Current physical activity guidelines [5] specify a minimum dose of physical activity to achieve health benefits, encourage increasing this dose to yield greater benefits, and warn that beyond an undefined threshold, risk of musculoskeletal injury [9] or adverse cardiac event [10] outweigh positive health gains. Characterization of the appropriate progression to complete the minimum dose to achieve specific health benefits and identification of a maximum dose that will not cause adverse effects, however, has been limited by a paucity of data [11]. Additionally, effective application of these guidelines is complicated by considerable heterogeneity in individual fitness and physiological responses to physical activity [12-14]. Given the complexity and ambiguity of the individual dose-response relationship between physical activity and fitness, it is not surprising that many physicians have undervalued the prescription of physical activity as part of routine clinical care [15,16], and many individuals do not achieve the minimum recommended physical activity level [17,18].

Web-based interventions offer an increasingly popular approach to dispense physical activity and health behavior guidance [19-24]. Internet access enables programs to reach large numbers of adults at reduced cost compared to face-to-face meetings, provides convenient access to health information, and allows greater management of the intervention process [23,25]. Advances in computing technologies have also permitted the generation of tailored guidance, the personalization of health messages based on variables related to models of behavior change [26]. Tailored physical activity programs have been shown to produce positive improvements in physical activity levels. These increases, however, are typically modest, short-term, and vary greatly based on intervention features [27-30].

Objective assessment of physical activity and health outcomes may improve the efficacy of tailored, Web-based physical activity interventions [21]. Many programs have relied on surveys to assess physical activity habits [19,27], but underestimation or biased responses [31,32] have contributed to inconsistent physical activity outcomes [21]. Use of pedometers and activity monitors (AMs) to track and provide physical activity feedback has demonstrated positive effects on physical activity habits [33], but to date, few studies have used these devices to guide, tailor or evaluate physical activity interventions [34-39]. Further, monitors used in previous studies used technology that was unable to accurately quantify both the intensity and duration of walking and running [40] and physiologic responses such as heart rate. Many individuals have a difficult time identifying physical activity intensity [18]. For these individuals, objective assessment of physical activity intensity using heart rate may allow for more refined and adaptive feedback to help them achieve an effective and safe physical activity dose [1].

The development of an integrated technology platform that combines the accessibility of the Internet with objective data from the growing number of wearable devices may provide new opportunities for public health professionals and clinicians to promote physical activity and understand its relationship to health. Public health groups, for example, could partner with wearable device companies to track compliance to workplace wellness programs and award incentives. Similarly, physicians could create physical activity interventions tailored to specific patient populations, monitor adherence and physiological response, and make adjustments to the plan to more effectively achieve diverse health outcomes. As a first step towards realizing these opportunities, the objectives of this study were to (1) develop a personalized physical activity prescription (PPAP) app that combines dynamic Web-based guidance with multi-sensor AM data to guide and promote physical activity and (2) to assess the feasibility of using this system in the field.

## Methods

### PPAP App Development

A series of steps were completed to develop the PPAP app. Initial processes included selection of a target population and creation of a physical activity intervention framework. An algorithm was then written to personalize the physical activity framework for each participant and create daily physical activity prescriptions. This algorithm was encoded into a combined AM and dynamic Web app that created physical activity prescription files, managed AM data, and provided feedback to participants regarding their progress in the physical activity intervention. Administrative features were added to the app to improve monitoring of data quality and participant compliance. Descriptions of these development processes are presented in the following sections.

### Target Population

Adults with a low-risk for acute cardiovascular events during physical activity (asymptomatic men and women with ≤ 1 cardiovascular disease risk factor) and a sedentary lifestyle were the target population for the initial PPAP app. These individuals were selected because they (1) are likely to experience greater improvements in cardiorespiratory fitness (CRF) compared to individuals with moderate or high baseline CRF levels [41], (2) can safely pursue physical activity without medical examination or supervision [2], and (3) represent a large population that could potentially benefit from the PPAP app and improve various health outcomes. Prior to utilization of this PPAP app, potential participants would be screened via an in-person or telephone survey to assess their cardiovascular risk [42] and physical activity level [43]. Individuals with a moderate or high level of physical activity and those respondents categorized as moderate (≥ 2 or more cardiovascular disease risk factors) or high (have symptoms or diagnosed metabolic, pulmonary, or cardiovascular disease) risk for acute cardiovascular events during physical activity would not be considered part of the target population and would not be appropriate candidates for using this version of the PPAP app.

### Standard Physical Activity Intervention Framework

A twelve-week physical activity intervention plan based on American College of Sports Medicine (ACSM) training progression guidelines for sedentary, low-risk adults was adapted as a standard intervention framework [1]. In this intervention, different combinations of the components of a physical activity dose (ie, activity type, intensity, duration, and frequency) are incremented until the individual achieves the minimum weekly physical activity volume recommended by current Federal physical activity guidelines [5]. Activity type is restricted to walking and running due to the measurement capabilities of the multi-sensor AM used in combination with the app. Specific guidelines for weekly physical activity frequency, duration, and intensity are outlined (Table 1). Recommended frequency is set to a minimum of three sessions per week with an optional fourth day of activity. Each activity session includes three phases: warm-up (5 minutes), endurance (variable duration), and cool-down (5 minutes). Physical activity intensity is prescribed with target heart rate zones defined with the heart rate reserve method, the most accurate method of establishing target heart rate [44], with the Target HR Zone = ([HRmax - HRrest] · percent intensity) + HRrest, and where HR = heart rate (beats per minute [bpm]), HRmax = maximum heart rate (bpm), and HRrest = resting heart rate (bpm).

Progressive increases in physical activity duration and intensity are created to gradually increase the physical activity stimulus each week to allow positive physiological adaptations and improve health [1]. Because the target population has low baseline levels of physical activity and CRF, initial physical activity doses have a short duration and low physical activity intensity (ie, low target heart rate zone). This approach reduces the risk of aggressive overload of the body’s structures and may improve exercise adherence [1,46]. After the first week, increases in physical activity duration or intensity occur in an alternating biweekly manner by increments of approximately 20% and 5% of HRRe, respectively [1].

A 12-minute run/walk exercise field test (EFT) is included as the first activity session of the 12^th^ week. The purpose of this EFT is to explore the feasibility of estimating CRF and measuring post-exercise heart rate recovery (HRR) in unsupervised conditions outside of clinical environments. This information is needed to advance the utility of EFTs to screen and monitor coronary heart disease risk in large asymptomatic populations [45].

**Table 1:** The standard physical activity intervention framework created for the PPAP app.

Week	Frequency	Intensity	Duration^a^
	(Sessions/Week)	(%HRRe^c^)	(min)
1	3	40-50	20
2	3-4	40-50	25
3	3-4	45-55	25
4	3-4	45-55	30
5	3-4	50-60	30
6	3-4	50-60	35
7	3-4	55-65	35
8	3-4	55-65	40
9	3-4	60-70	40
10	3-4	60-70	45
11	3-4	65-75	45
12^b^	3-4	65-75	50

^a^Duration values do not include warm-up (5 min) and cool-down (5 min) periods.

^b^A 12-min walk/run exercise field test [45] is prescribed for the first activity session of the 12^th^ week.

^c^%HRRe: percent of Heart Rate Reserve

### PPAP Algorithm

**Figure 1:**
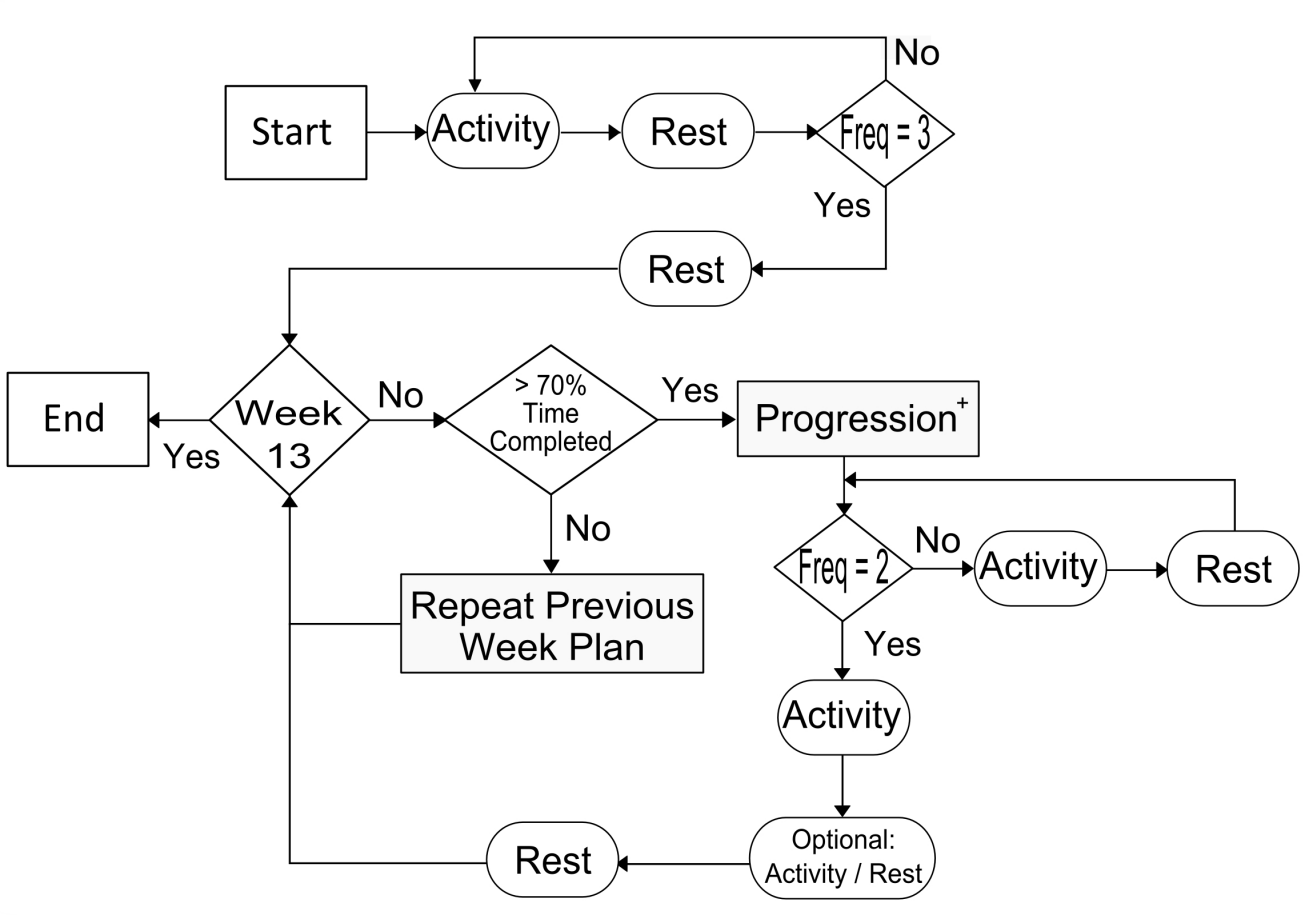
Illustration of the PPAP Algorithm used to decide the sequence of activity and rest sessions and the progression of exercise duration or intensity.

An algorithm was developed to create daily, personalized physical activity prescriptions from the standard physical activity framework for each participant. Daily and weekly physical activity doses completed by a participant are monitored and used to create the next dose prescription according to the rule-sets written for the PPAP algorithm (Figure 1). The algorithm begins at day one of an intervention and advances by day number, incrementing a counter each week. Within each week, physical activity frequency is queried to determine if a physical activity or rest session should be prescribed. The participant is presented an optional activity session on the sixth day of the week. If the optional session is selected, then its physical activity duration is added to the recommended total.

The algorithm includes two key personalization features: (1) the rate of progression and (2) adaptation of target heart rate zones. The rate of progression in the intervention is based on the individual’s compliance with the recommended physical activity sessions. Starting at the end of week two, the user’s weekly physical activity duration is calculated. If the user’s total duration is less than 70% of the recommended amount, the intervention does not advance to the next week’s prescription plan. Target heart rate zones are adjusted according to changes in each participant’s weekly average HRrest. This step enables the recommended physical activity intensity to be adapted to possible changes in the participant’s cardiovascular health that could occur in response to aerobic training over the time course of the intervention [47].

### Combined AM and Dynamic Web-Based Guidance

#### Overview

**Figure 2:**
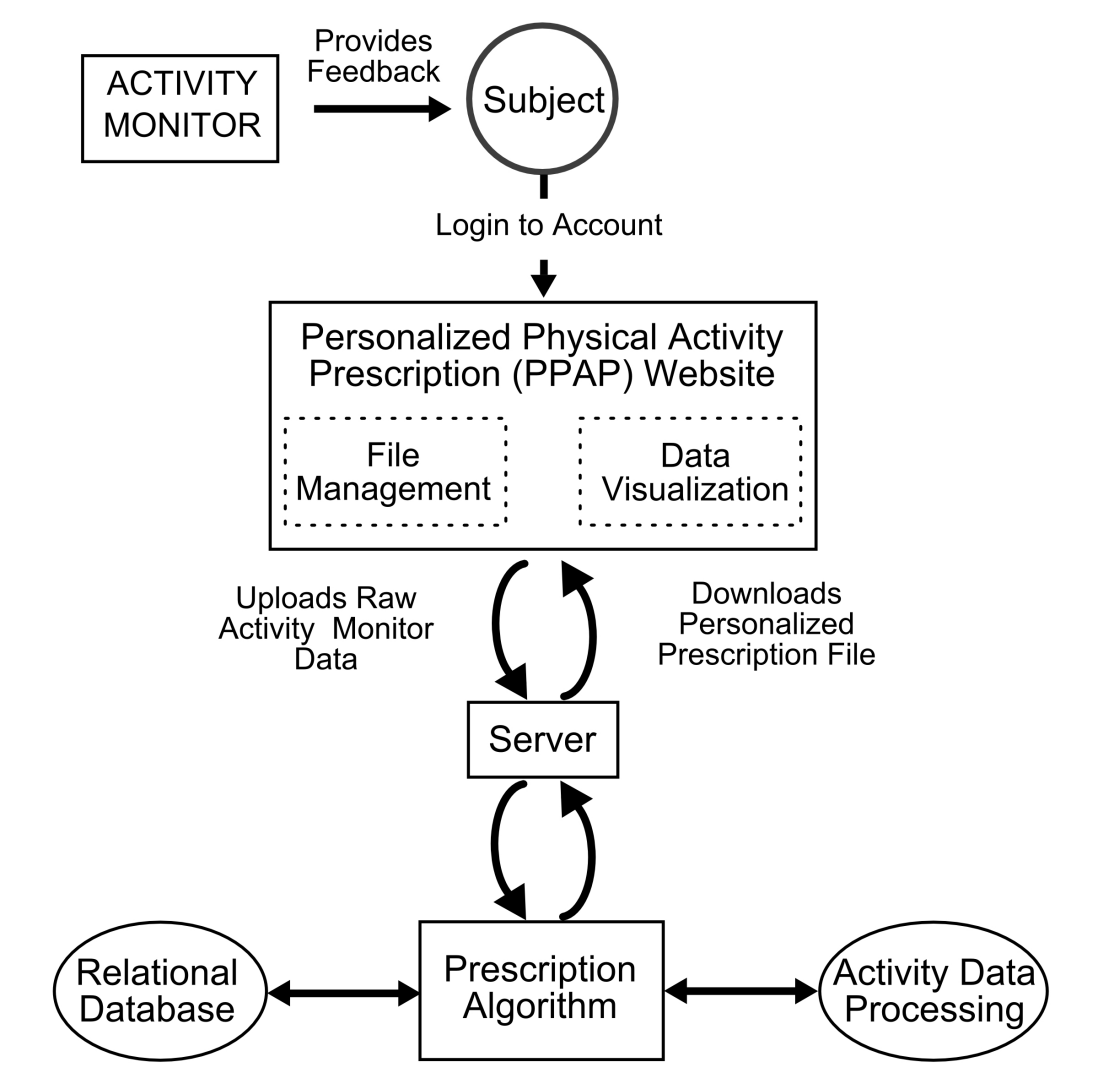
Diagram of the data-flow within the PPAP app.

A guidance program that combines AM data with a dynamic Web-based app was developed to generate personalized physical activity prescriptions, manage and store physical activity data, and provide interactive feedback to the participant. Data extracted from the participant’s AM directly influences the operation of the PPAP app (Figure 2). An overview of the multi-sensor AM, software components, and website features is presented in the following sections.

#### Multi-Sensor Activity Monitor

**Figure 3:**
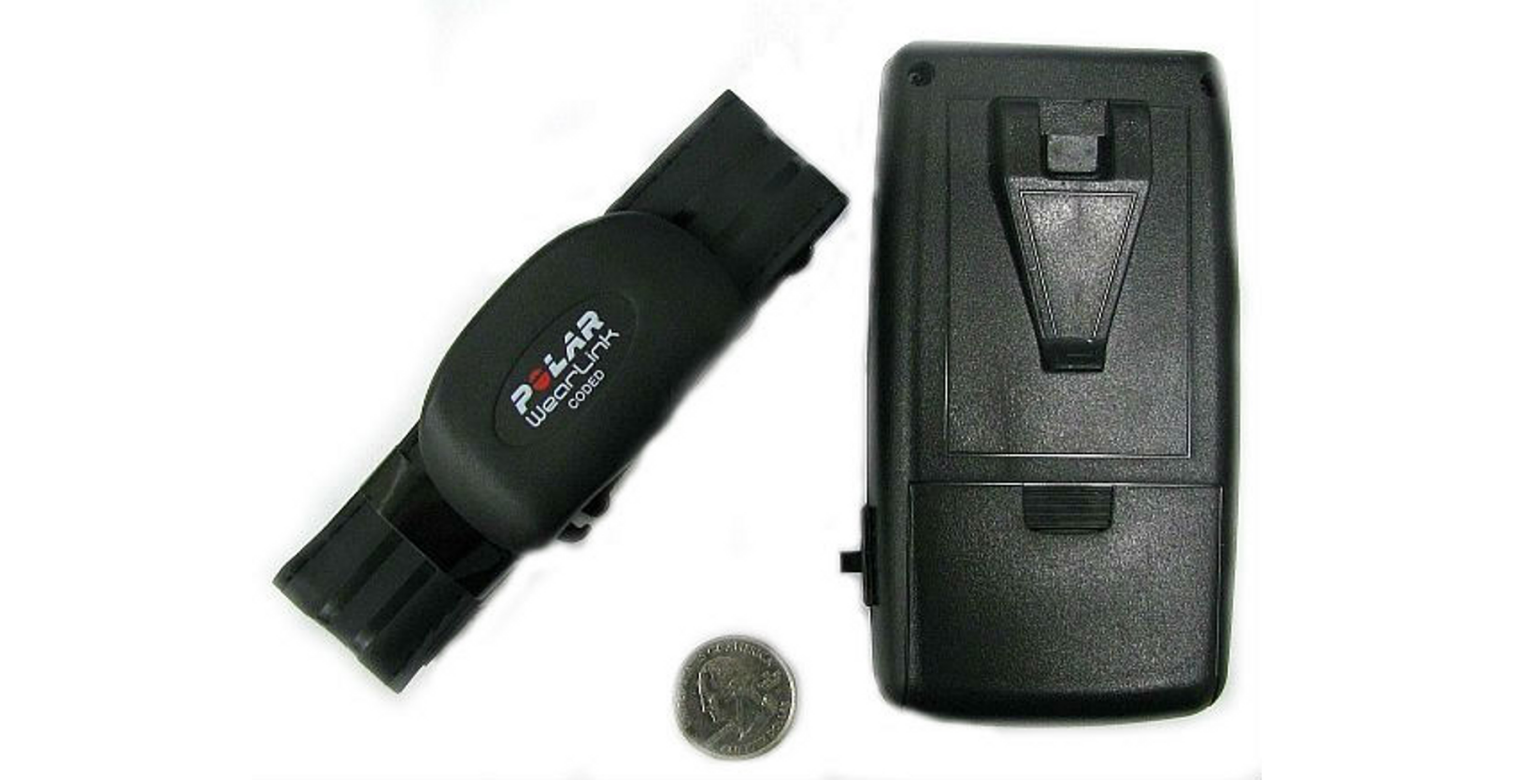
Illustration of the multi-sensor AM used to provide objective measures of resting heart rate and physical activity duration, frequency, and intensity to the PPAP app.

A custom, multi-sensor AM (Figure 3) was developed to obtain objective measures of physical activity and resting heart rate for the PPAP app. The custom AM contains a triaxial accelerometer (ADXL345 ± 8g amplitude range, Analog Devices, Norwood, MA) and a commercially available heart rate monitor (Polar Wearlink Coded 31 Transmitter and OEM module, Polar Electro Oy, Kempele, Finland). Acceleration [48] and heart rate data [49] using these sensors have been previously shown to be accurate and reliable. The AM operates for up to 70 hours on single battery charge and can record approximately 728 hours of binary acceleration and heart rate data to a 1 GB micro-SD card, a sufficient data storage capacity for field-based physical activity surveillance studies.

The AM has two modes of operation: a default mode and a resting heart rate mode. During default mode, the AM records continuous triaxial acceleration and heart rate data to an “activity” file. These data are used to determine physical activity frequency, duration and intensity. When resting heart rate mode is initiated, ten minutes of instantaneous heart rate data are recorded to a “heart rate” file, and data from the middle five-minute interval are averaged to determine a daily HRrest value. Quantification of these AM data enables physical activity prescriptions to be personalized for each participant.

The AM also provides the participant with a tool to monitor his or her progress and compliance during an activity session. Daily physical activity prescriptions are written to a binary data file that is downloaded to the AM memory card. When the AM is turned on, the file contents (specific physical activity duration and target heart rate zone limits) are read and used to trigger visual feedback in the form of small lights. Two blinking lights on the top of the AM are illuminated for five seconds to inform the participant of the end of warm-up, endurance, and cool-down phases of the activity session. During the endurance portion, the AM computes a five beat moving average of instantaneous heart rate, and a sequence of lights are turned on if the average heart rate is above or below the target heart rate zone. This feedback is designed to encourage participants to avoid under- or over-estimation of moderate and vigorous intensity levels.

#### PPAP App Software Components

Free, open-source software components were chosen to implement the PPAP algorithm and create a dynamic website [50] that would vary according to user parameters. Open-source software was selected to capitalize on continuous performance and security developments, and to allow experienced contractors or software engineers to manage system administration. The selected LAMP (an acronym derived from the first letter of each software component) solution stack included: a Linux operating system (Ubuntu LTS version 12.04 (Precise Pangolin), Canonical Ltd, London, United Kingdom), Apache Web server (version 2.2, Apache Software Foundation, Forest Hill, MD), MySQL database management system (version 5.1.37 | Ubuntu, Oracle, Redwood City, CA, USA), and PHP scripting language (version 5.2.10, The PHP Group). In addition to these core software, format and layout of the website was completed using HTML, cascading style sheets (CSS), and JpGraph, an object-oriented graph library for PHP (version 3.0.7, Asial, Tokyo, Japan). Processing of AM files is performed using custom scripts written in MATLAB (version r2010a, The Mathworks, Natick, MA).

#### PPAP App Dataflow

Server-side scripting is used to control dataflow, manage user-specific Web content, and create physical activity prescription files (Figure 2). The server was designed to return responses to three specific participant requests: (1) account creation/login, (2) AM file management, and (3) data visualization. The server contains 2 TB of storage, and load testing was performed to ensure it could quickly manage simultaneous requests from a minimum of 50 participants without loss of data. PHP functions (“Prescription Algorithm”) process requests and coordinate actions via “Activity Data Processing” or “Relational Database” software. Interpretation of PHP functions by the server generates the resulting Web page for the participant.

The system administrator coordinates account creation and login. Following eligibility screening and baseline fitness testing, an account is created for each participant and populated with physiologic information (ie, HRrest and HRmax values) to customize target heart rate zones. This action triggers an automated email to the participant with instructions to select unique login and password information. Account information is encrypted to protect the participants’ anonymity from both external attacks and administrative oversight.

AM file management comprises the upload of new AM files and download of personalized physical activity prescription files. PHP functions eliminate empty or repeated files, identify file type (“activity” or “heart rate”), and initiate appropriate data processing scripts for each file uploaded to the server. Custom MATLAB functions complete a series of steps to process raw data into computable structures for the PPAP algorithm. First, binary files are converted to integer and character variable arrays. Data quality is then evaluated to assess AM functionality and subject compliance. Activity data are excluded from the PPAP algorithm if no heart rate data are recorded or the average heart rate during the physical activity session is < 80 bpm. Resting heart rate data are filtered with a 10 beat moving average to remove aberrant beats or noise. Heart rate data are not used for the calculation of weekly average resting heart rate if > 20% are filtered or if the file is less than 7.5 minutes in length. Finally, relevant processed data features such as physical activity duration, calibrated triaxial accelerations, heart rate, and resting heart rate are exported to text files labeled with unique user and file identification keys for storage in the relational database. The relational database management system coordinated the storage and retrieval of data in response to PPAP algorithm and user driven queries. The database also contains physical activity duration and intensity limits based on the standard physical activity intervention framework (Table 1). PHP functions based on the PPAP algorithm (Figure 1) retrieve user-specific physiologic data (target heart rate zone), intervention date, and standard plan constraints to create personalized physical activity prescription files for the participant to download to the AM.

**Figure 4:**
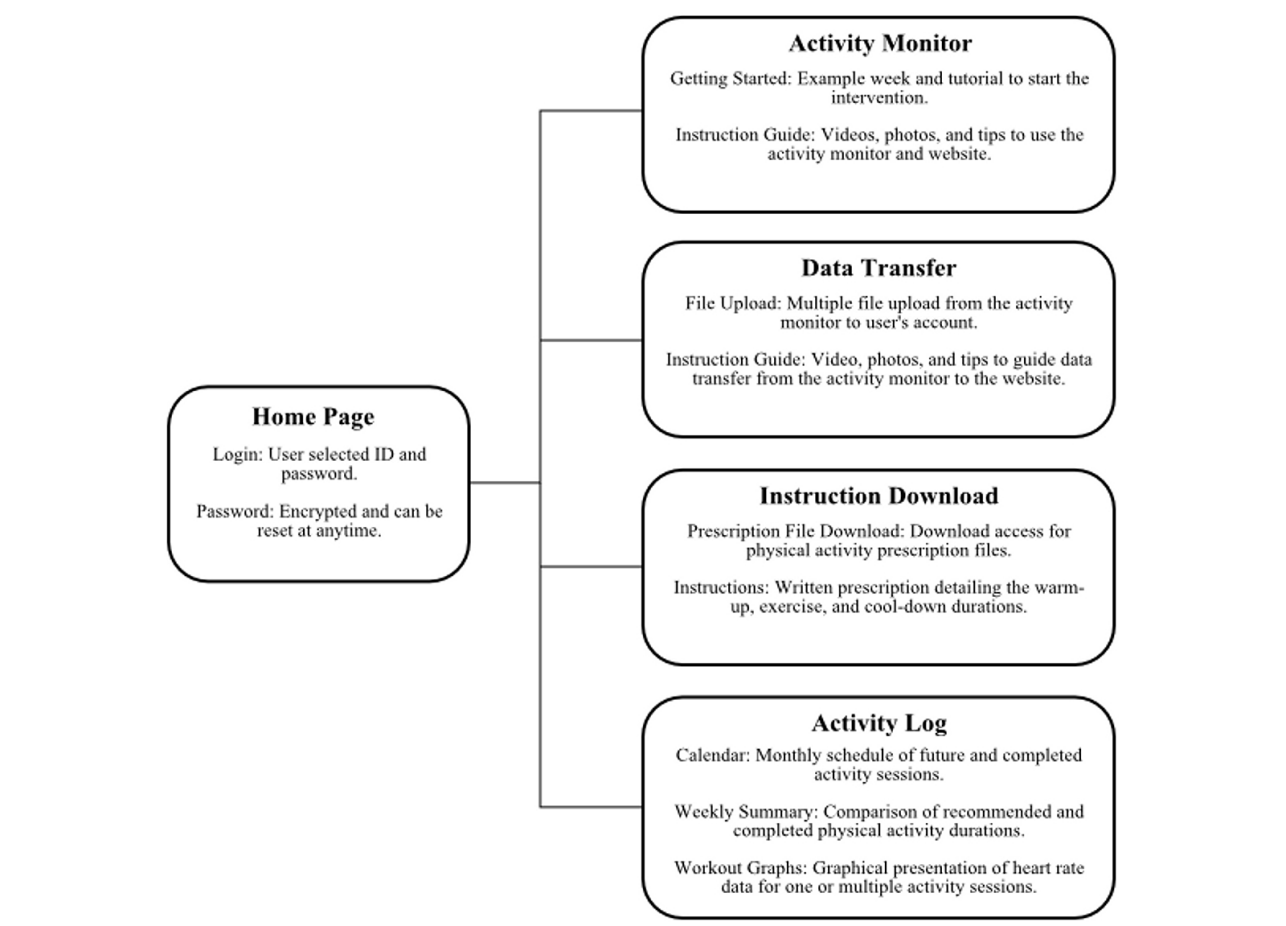
Description of the content and interactive features of the PPAP website.

Lastly, an interactive Web environment was developed to provide resources and data visualization to guide users during the physical activity intervention (Figure 4). Instruction manuals, tutorial videos, and frequently asked questions and answers were created to provide guidance regarding the operation and maintenance of the AM. Data transfer and instruction download menus provided users with easily accessible tools to upload and download AM and physical activity prescription files, respectively. Participants could also compare completed and recommended physical activity data in multiple interactive formats. A calendar displayed both a monthly schedule of future physical activity recommendations to assist with weekly planning and a reference to track completed physical activity sessions. Daily and weekly physical activity duration totals were also summarized in a tabular format. Participants could use this feature to track improvements in weekly physical activity duration and monitor their progress in the intervention. Further, participants could graph up to three completed individual physical activity sessions to compare changes in heart rate response during activity.

#### Administrative Monitoring Features

A series of quality assurance and administrative monitoring features were created to manage errors resulting from the complex interactions between the subject, AM, and PPAP app (Table 2). Ideally, participants uploaded AM files to the PPAP app on a daily basis. Perfect compliance with this schedule, however, was not expected due to possible Internet/computer access and time constraints. Management of delayed upload or missing data, therefore, was a primary concern for accurate personalization of physical activity prescriptions. Missing resting heart rate and activity data resulted in the insertion of a ‘null’ value or zeros in the database, respectively. If the subject uploaded the file on a different date, the filename and timestamp were used to determine allocation of the data to the correct intervention date in the database. If multiple resting heart rate files were uploaded on a single day, then the first file that passed quality assurance checks was saved. Multiple activity files, however, were concatenated so that the user received credit for all activity sessions completed on a given day. Generation of an optional physical activity session (Figure 2) required the subject to complete no more than two consecutive days of physical activity. If the optional file was not downloaded, its duration was not added to the weekly total. Calculation of completed weekly physical activity duration occurred at the end of the intervention week. Consequently, any activity files uploaded after the start of a new week were not included in the adherence check (Figure 2).

Additionally, the pattern of file management provided a method for study administrators to communicate and track participant compliance with the PPAP app. Automated emails were sent to both the participant and study administrator following account creation, at the start of the physical activity intervention, one day prior to the EFT, and at the conclusion of the intervention. These emails contained instructions, helpful links, and contact information for the study administrator. In addition, missing resting heart rate data for three consecutive days or the lack of file uploads for seven consecutive days triggered an automated email reminder and survey (Table 2), respectively. The survey was constructed to identify any possible problem with the participant’s compliance with the intervention (e.g. injury, illness, travel, technical difficulties). The system administrator also received daily logs of file upload and quality assurance checks for each participant. These logs enabled the administrator to identify errors with file transfers and AM malfunctions such as a faulty heart rate monitor battery.

**Table 2:** Remote administrative data monitoring features in the PPAP app.

	Frequency	Error type	Corrective action
**Heart rate**			
	Single day	No data	Insert null into database.
		Late upload	Extract database location from filename.
		Multiple files	Use the first file that passes quality checks.
	Three days	No data	Insert null into database and email reminder.
		Late upload	Extract database location from filename.
**Activity**			
	Single day	No data	None.
		Late upload	Extract database location from filename and create prescription using these data.
		Multiple files	Concatenate physical activity duration data.
	Two days	Optional day	Recommend rest for optional day.
**Prescription**			
	Single day	Optional day	Add prescription to total if file is downloaded.
	Multiple days	No download	Email notification to study administrator.
**All files**			
	Missed 7 days	No data	Email user to identify problem source.

### PPAP Feasibility Study

A feasibility study was completed to ensure that the PPAP app could administer a 12-week physical activity intervention and that website features were intuitive, easy to navigate, and motivational. Potential subjects were screened via a telephone survey to assess their physical activity level and risk for acute cardiovascular events during physical activity. Two apparently healthy, sedentary men who passed the screening and satisfied the target population characteristics were recruited from the Sacramento, CA area, and enrolled in the study. The University of California, Davis Institutional Review Board approved the protocol, and both subjects gave written informed consent.

#### Protocol

##### Overview

Each subject completed a preliminary session, baseline resting heart rate measurements, and 12 weeks of physical activity training.

##### Preliminary Session & Baseline Resting Heart Rate Measurements

During the preliminary session, subjects were given a multi-sensor AM, an account on the PPAP app, and an orientation to AM and website functions. Baseline resting heart rate measurements were completed for at least five days at the subject’s home to establish a HRrest value for the PPAP algorithm. To complete a resting heart rate session, the subject would don the AM, initiate the resting heart rate mode, and lay supine for 10 minutes immediately after waking. If the resting heart rate data were not uploaded to the server, HRrest was set to 65 bpm to avoid algorithm errors.

##### Physical Activity Intervention

Subjects were provided with 12 weeks of personalized physical activity guidance via the PPAP app. Subjects were instructed to complete daily resting heart rate and prescribed physical activity sessions while wearing the AM and to regularly upload AM files from these sessions to the PPAP app. In addition, subjects received instructions to perform a 12-minute run/walk EFT at the start of the 12^th^ week.

#### Data Analysis

Weekly recommended and completed physical activity volume was calculated as mean training impulse (TRIMP). TRIMP captures both the duration and heart rate of a walking or running session with TRIMP = exercise time (min)· HRr · e ^(1.92 · HRr)^ , where HRr = heart rate reserve ratio, “e” is an exponential function, and 1.92 is the appropriate scaling coefficient for men (it would be 1.67 for women) [51]. The recommended TRIMP was calculated using the average of the target heart rate zone end points. For example, if the recommended % HRRe was 40-50%, then the heart rate reserve ratio (HRr) was 0.45. For completed physical activity sessions, HRr was calculated as the average heart rate during the endurance portion divided by average HRrest for that day.

## Results

**Figure 5:**
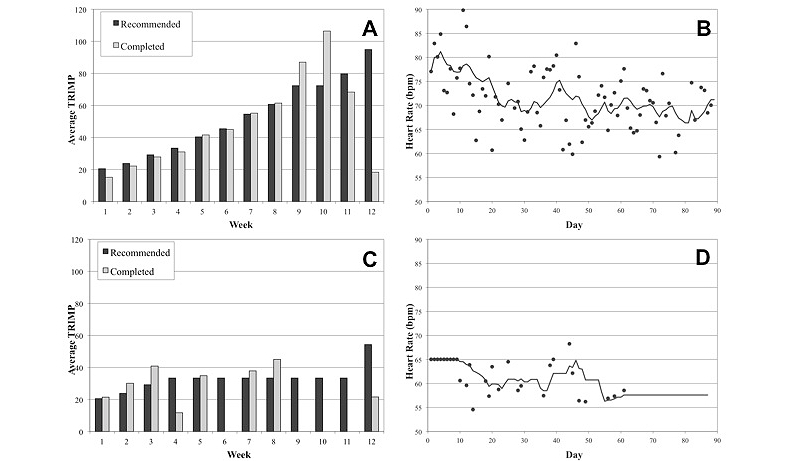
Comparison of physical activity and resting heart rate data recorded for two subjects during the 12-week PPAP application feasibility study. Completed TRIMP values for S1 demonstrated strong adherence to the recommended physical activity prescription (A). Observed (filled circles) and 10-day moving average (solid line) HRrest for S1 demonstrated a downward trend during the 12-week intervention (B). S2 progressed into week 4 of the intervention, but he did not achieve 70% of the recommended weekly physical activity duration for the subsequent weeks (C). Despite poor adherence to the program, the intervention progressed in week 12 to initiate an EFT. Email reminders were sent at the start of week 5 and 8, and S2 had limited computer access for weeks 9-12. A reminder was not sent for week 6 because S2 uploaded heart rate files to the server (D).

The two subjects (S1 and S2) demonstrated divergent adherence patterns to the PPAP intervention. S1 demonstrated excellent adherence to recommended physical activities progressing into week 10 of the standard physical activity training plan. Due to a late file upload after the end of week 9, S1 did not pass the compliance checkpoint (< 70% duration) resulting in the repeated prescription of week 9. He also did not pass the compliance check for week 11; however, the intervention was advanced to week 12 to initiate the prescription of the 12-minute run/walk EFT. S1 completed the 12-minute run/walk EFT achieving a distance of 2253m or an estimated peak oxygen consumption rate (V̇O_2peak_) of 48.5 ml·kg^-1^·min^-1^[45]. Comparison of recommended and completed TRIMP values (Figure 5A) indicated that S1 was able to achieve the recommended duration and intensity until weeks 9 and 10. At this point, S1 completed the appropriate physical activity duration, but his average heart rate during the endurance portion of the activity session was greater than the recommended target heart rate zone. Average resting heart rate data (Figure 5B) demonstrated a downward trend over the 12 weeks.

S2 completed recommended physical activity prescriptions during the first three weeks of the intervention, but his inconsistent participation prevented his progression past week four of the standard physical activity training plan (Figure 5C). S2 received email reminders during weeks five and eight of the intervention. Despite not completing physical activity during week six, S2 did upload resting heart rate data (Figure 5D), which prevented the generation of additional reminders. S2 did achieve the recommended TRIMP for week seven; however, he did not surpass the compliance checkpoint (< 70% duration) to trigger progression in the intervention. Response to a participation survey sent to S2 at week 9 indicated S2 had a lack of computer access for weeks 9-12; however, S2 did complete the 12-minute run/walk EFT at the start of week 12 achieving a distance of 2414m or an estimated V̇O_2peak_ of 51.4 ml·kg^-1^·min^-1^[45].

## Discussion

### Principal Findings

Advances in technology have resulted in an unintended, systematic removal of physical activity from daily life [52], yet these same technologies offer new opportunities to encourage people to lead active lifestyles. Improved activity monitors enable objective assessment of an individual’s health response to physical activity and have been primarily used to provide open-loop feedback to individuals regarding their heart rate and activity completed. These devices provide the potential to create closed-loop systems that acquire physiological signals which can be used to monitor an individual’s activity and the physiological response to this activity. This information can then be used to tailor future physical activity recommendations. Therefore, we developed a Web-based app that combines a dynamic interface with quantitative multi-sensor AM data to personalize physical activity prescriptions. Although limited in sample size, the dichotomy of participation of two sedentary adult men in a feasibility study enabled thorough testing of key program features in the field including: adaptation of a standard physical activity framework to create personalized physical activity guidance, creation of interactive physical activity feedback and website features, and remote administrative monitoring of data quality and subject compliance. Further, the trial demonstrated that an EFT could be completed without supervision, a potentially important advance for using a technology-based approach to assess cardiovascular disease risk in asymptomatic populations.

### PPAP App Development

A main contribution of the PPAP app is the tailoring of physical activity guidance according to principles of physical activity training (ie, overload, progression, and specificity) [46,53]. Contrary to this approach, many Web-based physical activity programs use tailoring strategies based on theories of behavioral change to determine the amount of physical activity recommended to the participant [19-23]. Increasing physical activity without consideration of the participant’s physical activity history, however, could increase fatigue or risk for musculoskeletal injury [46], complications that can result in temporary or permanent stoppage of a physical activity program [54]. Individual response to exercise training is also highly heterogeneous [12-14], thus many individuals may be discouraged from physical activity participation due to a lack of observable improvements in fitness or health. The PPAP app can serve as an initial framework for clinicians, researchers, and public health professionals to remotely investigate and precisely characterize these barriers to regular physical activity participation. Physical therapists, for example, could use the survey tools in the PPAP app to identify individuals who experience a musculoskeletal injury, follow-up with the participant in clinic to make a formal diagnosis, and characterize precisely the amount of physical activity training that resulted in injury. Similarly, simple alterations to the PPAP algorithm could enable the creation of customized physical activity interventions to be used in conjunction with health coaching programs to better investigate behavioral components of physical activity programs [55].

The multi-sensor AM used in combination with the PPAP app enabled accurate assessment of subject compliance with recommended physical activity doses during the intervention. Previous Internet-based interventions have used step counts from pedometers [34,38] or “activity counts” and “PAM scores” from uniaxial AMs [35-37,56] to guide tailoring and provide feedback to participants. Pedometers, however, are unable to capture physical activity intensity, frequency, or duration [57], and uniaxial AMs lack accuracy to measure running or other activity types [58]. These device limitations may have affected the accuracy of physical activity recommendations and evaluation of the efficacy of these interventions to increase physical activity [35-37]. The multi-sensor AM used in this study was designed to accurately measure various speeds of walking and running [48]. While the lack of activity type diversity prescribed by the PPAP app could negatively impact program adherence [1], gait activities were advantageous as they required little skill or equipment, involved large muscle groups, and triggered positive cardiovascular adaptations [46].

### PPAP Feasibility Study

Objective measures of physical activity recorded by the multi-sensor AM were also beneficial in educating subjects about their physical activity intensity. Current physical activity guidelines use relative terms (e.g. light, moderate, or vigorous) to describe the recommended physical activity intensity to achieve health benefits [5], yet many adults have difficulty interpreting this language resulting in an underestimation of intensity levels [18]. To overcome this limitation, we programmed the AM to use instantaneous heart rate and personalized target heart rate zones to provide visual feedback to the user during the activity session. In addition, workout charts comparing heart rate data obtained from different physical activity sessions allowed subjects to link periods of high intensity with changes in their physiological response, giving them the ability to self-monitor their physical activity intensity in subsequent activity bouts. S1 reported favorably about these features, which supports the agreement between his completed and recommended TRIMP values. As suggested by Hurling [35], charts and calendars comparing recommended and completed physical activity may have motivated this subject to increase his physical activity levels and set goals. Future quantitative studies, however, are necessary to monitor metrics such as how often and how long these interactive features are used to evaluate the potential effect of this material on physical activity [29].

Administrative features incorporated into the PPAP app were effective in monitoring data quality, subject adherence, and AM functionality. The study administrator independently verified rejection of activity or heart rate files due to data quality errors. The most common cause for rejection of heart rate data was filtering > 20% of the recorded data, which could occur if the heart rate monitor did not have adequate contact with the subject’s chest. Email reminders, an effective tool to encourage exposure and reduce attrition in Internet-based interventions [59,60], sent to S2 also resulted in temporary increases in physical activity and website interaction. The subject’s response to the seven-day survey at the start of week 9 also allowed the study administrator to identify limited computer access as the source of program attrition. Unlike other intervention programs [29], the email reminders and surveys in the PPAP app were based on user interaction with the website rather than a set schedule and did not require administrative action, which improves the feasibility of using this approach for large-scale population based interventions.

While the feasibility study demonstrated the functionality of the PPAP app in the field, the generalizability of these findings to a larger population are unclear. Both subjects in the study were adept at using technology and had daily access to the Internet at the start of the intervention (S2 did not have computer access for the end of the intervention). While data from the Pew Research Center indicate that 87% of American adults used the Internet in 2014 [61], offline adults, who are typically older (> 65 years), in the lowest socio-economic bracket, or have less than a high school education [62], would not benefit from using the PPAP app. Web-based interventions may also be ineffective for promoting physical activity in global communities where Internet access is limited [63]. Further, participants may be hesitant to use the PPAP app due to concerns with electronic data privacy [64]. While it may not be possible to address these general technical limitations, the PPAP app does include: (1) various instructional guides to help bridge technology education gaps, (2) a prescription algorithm and technology framework that can be implemented on smartphones or as a phone messaging service to reach global communities where cell phone access is more universal [63], and (3) a complex data encryption routine to provide safe data storage.

### Next Steps

The PPAP app provides a foundation to use a technology-based approach to physical activity promotion; however, there are several challenges that must be addressed to make the program applicable to a range of populations. First, an efficacy trial needs to be performed with sufficient sample size to establish the generalizability of the PPAP app in a diverse population. A randomized control trial comparing the PPAP app to the ACSM physical activity intervention can then be conducted to assess the effect of the program on CRF and HRR in sedentary adults. Second, screening for sedentary physical activity behaviors via telephone survey does not provide objective measures of baseline fitness. It is possible that a spectrum of CRF levels exist within this classification. As a consequence, the low duration and intensity of the physical activity doses at the beginning of the standardized program may not be of sufficient magnitude to stimulate physiological adaptations in some individuals, and this population would experience smaller improvements in CRF compared to individuals with lower baseline fitness. One approach to mitigate this difference could include a baseline physical activity assessment period with the AM to establish an initial dose for each individual prior to entering a progression period in the intervention. Third, health outcomes could be influenced by performance of physical activities at work, home, or transportation that were not included in the intervention. As the multi-sensor AM is not currently capable of detecting multiple activity types, interactive physical activity surveys could be incorporated into the website to provide additional feedback to supplement AM data. Fourth, the PPAP app is limited in its generalizability to other subject populations. Incorporation of additional physiological measures such as heart rate variability or blood glucose readings may allow for customization of the PPAP intervention for a more aggressive physical activity intervention that could maximize CRF in recreational runners [65] or that could maintain glucose homeostasis in diabetic individuals, respectively. Lastly, integration of behavioral modification theories into website features and physical activity messages should be considered as a possible approach to improve intervention adherence [66].

### Conclusions

This study describes the development and testing of a PPAP app that integrates objective AM data with dynamic Web-based guidance to provide a closed-loop approach to promote physical activity in sedentary adults. Results from a 12-week feasibility study demonstrated the ability of the PPAP app to create daily, personalized physical activity sessions, generate interactive Web-based feedback, and remotely monitor participant compliance and AM functionality with minimal investment of time and staff resources. The selection of ubiquitous software components as the foundation of the PPAP app allows healthcare professionals and researchers to replicate this technology framework, adapt the physical activity prescription algorithm, and personalize physical activity interventions to achieve health outcomes in a variety of subject populations. As advances in physiologic monitoring improve, patterns and thresholds for musculoskeletal injury risk and coronary heart disease risk reduction therapies can be incorporated into new interventions in the PPAP app, thus enabling the development of personalized physical activity prescriptions that minimize injury risk, maximize CRF, and reduce risk factors for coronary heart disease.

## Abbreviations

ACSM: American College of Sports Medicine
AM: activity monitor
bpm: beats per minute
CRF: cardiorespiratory fitness
EFT: exercise field test
HR: heart rate
HRr: heart rate ratio
HRR: heart rate recovery
HRRe: heart rate reserve
HRmax: maximum heart rate
HRrest: resting heart rate
PPAP: personalized physical activity prescription
TRIMP: training impulse
S1: subject 1
S2: subject 2
V̇O_2peak_: peak oxygen uptake
